# Supplementary material for: MetaRibo-Seq measures translation in microbiomes
Source: Nat Commun. 2020 Jun 29;11:3268. doi: 10.1038/s41467-020-17081-z (PMC7324362; doi:10.1038/s41467-020-17081-z)
Supplement: Supplementary file 10 — Supplementary Data 7 [file 41467_2020_17081_MOESM10_ESM.zip › File2/Confidence_High_Taxonomy/24190_out.krona.html]

Javascript must be enabled to view this page.

members
magnitude
magnitudeUnassigned
count
unassigned
taxon
rank

24190\_out

2

2
superkingdom
2

phylum
1239
2

1
class
186801

order
186802
1

family
186803
1

1
841
genus

1
species
166486

SRS143372\_contig\_number\_contig-100\_16914.16915

class
91061
1

1
186826
order

family
1300
1

1
1301
genus


SRS149186\_contig\_number\_94
1
1313
species
